# Association between exposure to smoke from cooking fuels and anaemia among women of reproductive age in Ghana

**DOI:** 10.1038/s41598-024-66602-z

**Published:** 2024-07-08

**Authors:** Samuel Akwasi Adarkwa, Michael Safo Oduro, Anthony Kwame Morgan, Seth Arhin-Donkor

**Affiliations:** 1 Department of Statistical Sciences, Kumasi Technical University, Kumasi, Ghana; 2 Pfizer Worldwide Research and Development, Pharm Sci and PGS Statistics, Groton, CT 06340 USA; 3 Department of Geography and Rural Development, Kwame Nkrumah University of Science and Technology, Kumasi, Ghana; 4 Humana Inc., Market Finance Analysis - Sr - Prd - Regional, Louisville, KY 4020 USA

**Keywords:** Anaemia, Cooking fuels, Indoor air pollution, Smoke, Women of reproductive age, Energy and society, Sustainability, Environmental impact

## Abstract

In low- and middle-income countries, indoor air pollution (IAP) is a serious public health concern, especially for women and children who cook with solid fuels. IAP exposure has been linked to a number of medical conditions, including pneumonia, ischemic heart disease, stroke, chronic obstructive pulmonary disease (COPD), lung cancer, and anaemia. Around 500 million women of reproductive age (WRA) suffer from anaemia globally, with an estimated 190 million cases in sub-Saharan Africa (SSA). This study, which is based on prior research, investigates the relationship between IAP exposure and anaemia among WRA in Ghana. A diverse sample of 2,406 WRA living in Ghana were interviewed, of which 58.06% were anaemic and used high-pollutant fuels for cooking. Age, place of residence, region, education level, religion, ethnicity, wealth index, type of drinking water, type of toilet facility, and type of cooking fuels were all found to be significantly linked with anaemic state by bivariate analysis. Type of cooking fuels utilized, age, region of residence, and the type of residence were shown to be significant predictors of anaemia status using sequential binary logit regression models. The results emphasise the critical need for efforts to promote the usage of clean cooking fuel in an attempt to lower anaemia prevalence in Ghana. To reduce dependency on solid fuels for cooking, initiatives should promote the use of cleaner cooking fuels and enhance the socioeconomic status of households. These interventions could have significant public health effects by reducing the burden of anaemia and improving maternal and child health outcomes due to the prevalence of anaemia among WRA. Overall, this study sheds light on the relationship between IAP exposure and anaemia in Ghana and highlights the demand for focused public health initiatives to address this serious health problem.

## Background

Indoor air pollution (IAP) or household air pollution through the use of solid fuels for cooking is a serious public health issue in many low- and middle-income countries, including Ghana. In 2020, the World Health Organization (WHO)^1^ estimates that 3.2 million deaths were linked to household air pollution, with women and children being the severely impacted. Given that it is linked to several unfavourable pregnancy outcomes, including low birth weight and preterm delivery, as well as maternal health issues like respiratory infections, chronic obstructive pulmonary disease (COPD), and lung cancer, exposure to IAP is particularly harmful to Women of Reproductive Age (WRA)^2^. In developing countries, where anaemia is particularly prevalent, the use of biomass fuel for day-to-day energy is more frequent than in developed countries^3^, due to cost-related factors, ease of use, and accessibility^4^. According to the International Energy Agency (IEA), roughly 2.5 billion people in rural areas of developing countries depend on solid fuel as their major source of energy^5,6^. However, when solid fuel is not well burnt, large amounts of particulate matter, carbon monoxide (CO), hydrocarbons, free radicals, and chlorinated organics are produced^7^, making it one of the least efficient and unclean fuels in terms of combustion efficiency and cleanliness. Liquefied petroleum gas (LPG) and electricity, on the other hand, are ranked first since they are the most efficient and environmentally friendly^5^. Particulate matter (PM2.5 and PM10), CO, nitrogen dioxide (NO_2_), formaldehyde, benzene, butadiene, polycyclic aromatic hydrocarbons (including benzo[a]pyrene), and numerous other dangerous organic compounds are among the pollutants present in solid fuel smoke^8^. These chemical compounds have been associated with health disorders like pneumonia, stroke, ischemic heart disease, COPD, lung cancer, and anaemia^9^.

The two primary signs of anaemia, a serious public health illness, are low blood haemoglobin concentration (120 g/L for non-pregnant women) and a reduced red blood cell capacity to transmit oxygen^10^. Globally, anaemia affects about 500 million WRA and over 32 million pregnant women, making it one of the most serious public health crises affecting women^10,11^. Anaemia affected 1.8 billion people globally in 2019, according to the Global Burden of Disease (GBD) Study (age-standardized point prevalence: 23.18%)^12^. Anaemia in WRA is a major public health issue that is associated with negative health, nutrition, social, and economic outcomes^13^. Sub-Saharan Africa (SSA) is a particularly sensitive region to anaemia, with an estimated 190 million cases in the sub-region^14^. Anaemia raises the risk of pregnancy-related morbidity and death, as well as poor pregnancy outcomes including low birth weight and limited development in infants^10,15^. Pregnant women had a greater prevalence of anaemia (36.5%) than non-pregnant women (29.6%)^15^. Lower- and middle-income countries (LMIC) have the highest anaemia prevalence (43.7%) among reproductive-aged women, followed by low-income countries (38.8%)^15^. Anaemia is the top risk factor for impaired cognitive function, poorer health-related quality of life (HRQOL)^16^, cardiovascular disease progression^17^, and increased all-cause mortality globally, according to a significant body of research^18^. According to epidemiological studies, the reasons for increased anaemia risk and changed anaemia-related blood cell parameters included age, nutritional deficiencies, inflammation, and air pollution^19–21^. Chemicals produced by burning solid fuel impede oxygen supply to tissues and causes systemic inflammation, which is mediated by inflammatory cytokines and can alter the erythropoietin process, resulting in anaemia^22,23^. There is significant global interest in reducing anaemia among WRA by half (sustainable development goal [SDG] indicator 2.2.3)^24,25^. Reducing the anaemia burden, particularly among WRA has birthed research into various factors associated with it and ways in which the situation can be averted.

In their study, Pathirathna et al.^26^ found no significant effect of solid fuel smoke exposure on the anaemic or non-anaemic condition of reproductive-aged non-pregnant Sri Lankan women. However, because the study was cross-sectional, the findings should be interpreted with care. The literature, on the other hand, is abundant with evidence that links the usage of biomass fuel to anaemia in WRA^27,28^. Furthermore, some studies sought to examine the ideal relationship between biomass fuel smoke exposure and anaemia^29,30^. Several studies have found a link between dirty cooking fuel smoke exposure and anaemia in children and pregnant women^31,32^. Likewise, He et al.^33^ found that utilizing solid fuel for cooking was positively correlated with prevalent anaemia risk in their investigation of the independent and combined effects of cooking fuel usage and socioeconomic status on anaemia risk as well as anaemia-related characteristics in rural China. There were also significant independent relationships between socioeconomic position (education and income levels) and frequent anaemia. He et al. discovered an association between low socioeconomic status and the use of solid fuel for cooking and the likelihood of progressive anaemia. These show the global correlations between the usage of solid fuel for cooking and the risk of anaemia.

 In Ghana, anaemia in WRA has decreased from 59% in 2008 to 42% in 2014. Tetteh et al.^34^ observed improvements in the wealth index of families as a result of a variety of characteristics including the mother’s age, education, usage of hormonal contraception, and body mass index (BMI) which partially explained the decline in anaemia among WRA over the years. The household wealth index, a measure of the family’s socioeconomic status (SES), the capacity of the household to obtain basic needs and healthcare services, and the ability of the household to provide for the needs of household members, was critical to the lowering prevalence of WRA in Ghana. Despite the seeming decline in anaemia prevalence, the 42% rate remains high, needing interventions at all levels. WRA have a disproportionate burden of undertaking home cooking chores within the Ghanaian culture, putting them at a higher risk of exposure to greater quantities of IAP produced by solid fuel stoves. However, there has been a paucity of research on the detrimental health effects of biomass smoke exposure in the Ghanaian context^35,36^, where a significant proportion of the population use firewood exclusively or in combination with LPG or kerosene for fuel/energy. The studies of Tetteh et al.^34^ and Armo-Annor et al.^37^ are the only known studies that explored solid fuel use-related anaemia among women. Even so, the study of Tetteh et al.^34^ considered the drivers of anaemia reduction among WRA, with less emphasis on the effect of cooking fuel use on anaemia risk, while focusing on The Eastern and Upper West regions of Ghana. Armo-Annor et al.^37^ also focused on women engaged in biomass-based fish smoking as their primary livelihood, implying that they have higher exposure rates compared with women who use solid fuel for cooking. The evidence so far presents a depth of research on fuel use and anaemia exposure among WRA in Ghana. Consequently, this study examines the relationship between cooking fuels smoke exposure and anaemia in reproductive-aged women in Ghana. We analyzed data from the Ghana Demographic and Health Survey (GDHS) 2014^38^—a nationally representative survey in Ghana. WRA (15–49 years) were included in the study. The findings of this study will add to the body of information on the relationship between cooking fuel and anaemia in WRA, as well as determine whether there are any special considerations in attempts to manage anaemia in WRA.

## Methods

### Data source

We used secondary data from the 2019 Ghana Malaria Indicator Surveys (GMIS), carried out between September 25 and November 24 2019 by the Demographic and Health Survey Measure Program^39^. The DHS MEASURE Program, which is available online for free upon request, was the source of the study's data. This application gives users access to a wide range of population demographics. Specifically, data on women’s anaemia statuses were acquired from the women’s file (aged 15–49). We collected and analysed data from the sample of women who completed the surveys. As secondary data was used in this study, ethical clearance was not applicable.

### Survey

The Ghana Statistical Service (GSS), the Ministry of Health (MOH), and the National Malaria Control Program of the Ghana Health Service undertook the survey, and the scope and methods of the GMIS have previously been made public. The Inner City Fund (ICF), and the Demographic and Health Surveys (DHS) Program offered technical assistance.

### Study variables

#### Dependent variable

**Figure 1:**
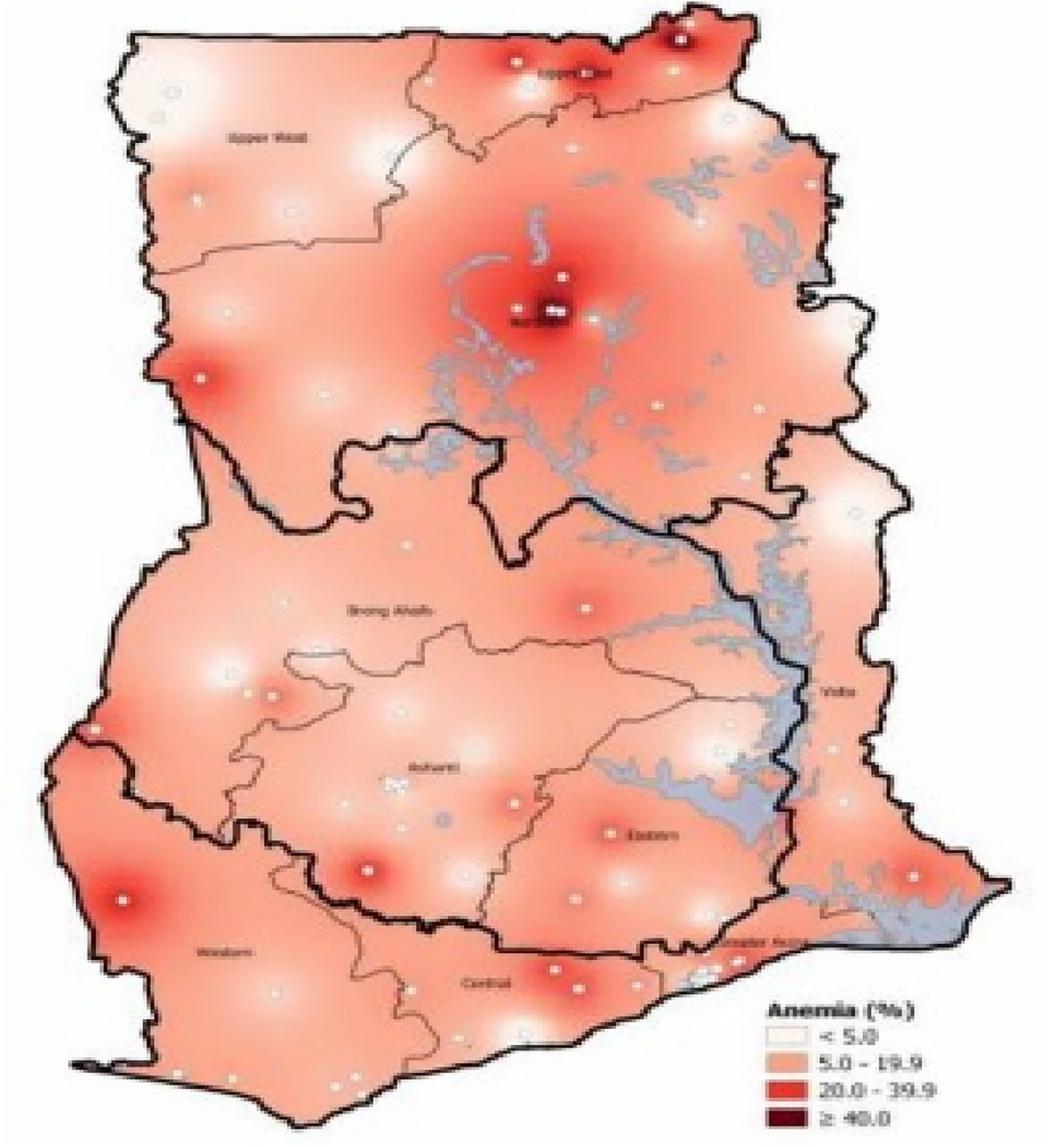
Distribution of anaemia prevalence among young children and non-pregnant women in Ghana. *Source*: Adopted from Wegmüller et al.^40^.

Using the information gathered on blood haemoglobin levels, pregnant and non-pregnant WRA were classified according to their anaemia status. To test for anaemia, women willingly gave blood samples, which included the collection of a little amount of blood via a finger prick in a microcuvette. A portable HaemoCue analyzer that ran on batteries was used for the analysis to measure the blood haemoglobin level. Each sample’s blood haemoglobin concentration was determined in g/dL following the on-site analysis. Kassebaum and Global Burden of Disease (GBD)^14^ criteria were applied to categorise the women’s anaemia status based on their haemoglobin (Hb) level. As a result, the women were divided into four categories: not anaemic (Hb 12.0 g/dL), mildly anaemic (11.0–11.9 g/dL), moderately anaemic (8.0–10.9 g/dL), or severely anaemic (Hb 8.0/dL). The women were further then divided into two groups: anaemic (mildly anaemic, moderately anaemic, and severely anaemic) and not anaemic. Figure 1 shows the distribution of anaemia prevalence among young children and non-pregnant women in Ghana^40^. Figure 1 is sourced from the study of Wegmüller et al.^40^.

#### Predictor variables

**Figure 2:**
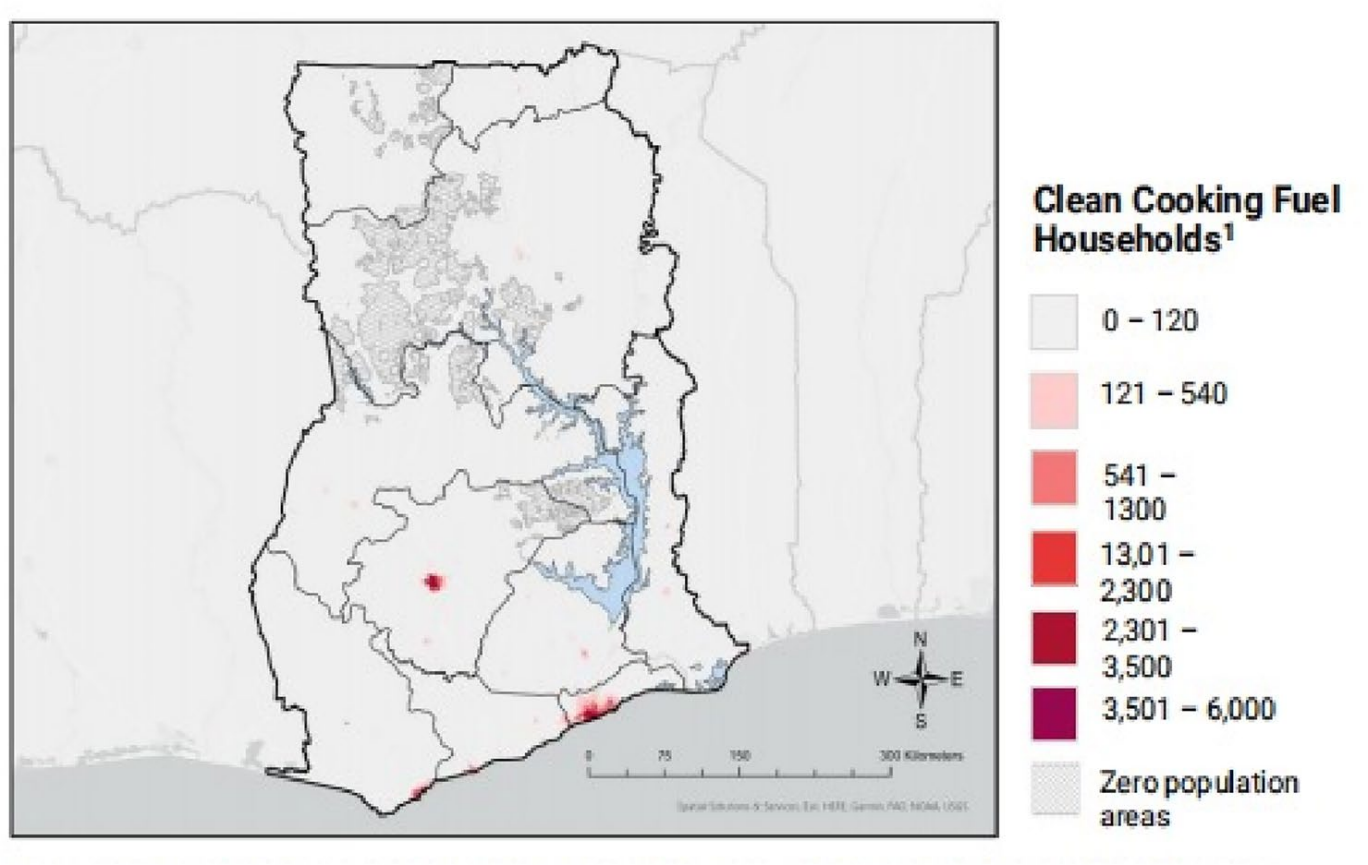
Distribution of household’s use of clean cooking fuel per 1 km^2^. Clean fuel includes electricity, LPG, natural gas and biogas. *Source*: Adopted from Clean Cooking Alliance^41^.

The kind of fuel utilised to generate domestic energy was the main independent variable in this study. Eleven (11) options (including electricity, LPG, natural gas, biogas, kerosene, coal/lignite, charcoal, wood, straw/shrubs/grass, agricultural produce, and animal dung) were given to the women when they were asked what their household’s primary source of cooking fuel was. These fuel types were then divided into three categories based on the volume of IAP produced: high-polluting fuels (wood, straw, plants, grass, animal dung, and agricultural crops); medium-polluting fuels (kerosene, biogas, coal/lignite, and charcoal); and low polluting fuels (electricity, LPG, and natural gas)^37^. Figure 2 shows the distribution of cooking fuel types used in Ghana^41^. Figure 2 was sourced from the study of Clean Cooking Alliance^41^. Additional independent variables included the place of residence (rural and urban) and wealth index (poorest, poorer, middle, richer, and richest). The wealth index, a composite score of a household’s total living conditions, was used as an indicator of economic status. This score was determined using the Principal Component Analysis (PCA) in which weights were applied to variables such as the ownership of durable assets, housing features, and service accessibility^42,43^. In this study, the DHS employed the wealth index to split questioned families into five “wealth quintiles” for comparison of the impact of economic status on various populations. The wealth index sets households on a continuous scale of relative wealth^43^. Other variables included the age of the mother (15–19, 20–24, 25–29, 30–34, 35–39,40–44, 45–49); maternal education (no education, primary, secondary, and higher); region (Western, Central, Greater Accra, Volta, Eastern, Ashanti Brong Ahafo, Northern, Upper West, and Upper East); ethnicity (Akan, Ga/Dangme, Ewe, Guan, Mole-Dagbani, Grusi Gurma, Mande, Other); and Currently Pregnant (No or unsure and Yes).

### Statistical methods

We employed both exploratory and inferential statistical methods to address the research questions in a comprehensive and insightful manner. Initially, the data was carefully examined to identify patterns and trends in the background characteristics of respondents, as well as the prevalence of anaemia. Descriptive statistics, including counts and proportions, were used to summarize the outcome variable and study predictors, and a contingency table was used to highlight the relationships between anaemia status and the various predictors. Also, a Chi-Square test of association was conducted to examine bivariate associations between anaemic status and the predictors for potential inclusion into the statistical models fitted. Furthermore, to avert multicollinearity issues, the Chi-square test was used to examine predictors that were associated with each other so as to retain the more informative and study the relevant variables. The next step involved fitting two sequential binary logit models. The first model considered cooking fuel as the primary predictor of the participant’s anaemic status. The second model, a multiple binary logit model, explored the adjusted impact of other demographic and socioeconomic variables on the prediction of anaemia status.

This model produces adjusted odds ratio estimates, as well as 95% confidence intervals and *p* values. All statistical analyses were carried out using the R software and conclusions drawn at a 5% level of significance.

### Ethics approval and consent to participate

As secondary data was used in this study, ethical clearance was not applicable. Therefore, the manuscript does not require a statement regarding the approval of experimental protocols by an institutional or licensing committee. Additionally, since no direct involvement of human subjects took place, informed consent was not required as an ethical consideration related to the study.

## Results

### Sample characteristics and prevalence of anaemia and types of cooking fuels

Descriptive summaries on the analytic sample are presented in Table 1. The study sample consists of 2,406 participants, with 41.94% being non-anaemic and 58.06% anaemic. Participants vary in age, education, ethnicity, and wealth. The majority live in rural areas (62.43%), with 37.57% in urban areas. Participants were stratified by age, with the largest group aged 25–29 (26.60%) and the smallest aged 45–49 (2.74%). The Northern region had the most participants (16.87%), while Greater Accra had the least (7.15%). Education levels varied, with 28.64% having no formal education, 45.22% with secondary education, and 4.36% with tertiary education. The largest ethnic groups were Mole-Dagbani (33.25%) and Akan (33.75%). Most participants were in the poorest (33.17%) and poorer (20.99%) wealth categories. The majority were not pregnant (91.23%), and most used high-pollution fuels for cooking (58.06%). In terms of drinking water, 87.41% used clean sources, and 12.59% used unclean sources. Regarding toilet facilities, 57.73% had clean facilities, and 42.27% had unclean ones.

**Table 1:** Descriptive statistics of study variables.

Variables	Levels/categories	Sample	Proportion (%)
Anaemic status	No	1009	41.94
	Yes	1397	58.06
Age in 5-year groups	15–19	90	3.74
	20–24	436	18.12
	25–29	640	26.60
	30–34	534	22.19
	35–39	431	17.91
	40–44	209	8.69
	45–49	66	2.74
Place of residence	Urban	904	37.57
	Rural	1502	62.43
Region	Western	240	9.98
	Central	214	8.89
	Greater Accra	172	7.15
	Volta	210	8.73
	Eastern	187	7.77
	Ashanti	247	10.27
	Brong Ahafo	230	9.56
	Northern	406	16.87
	Upper East	234	9.73
	Upper West	266	11.06
Highest educational level	No education	689	28.64
	Primary	524	21.78
	Secondary	1088	45.22
	Higher	105	4.36
Ethnicity	Akan	812	33.75
	Ga/Dangme	100	4.16
	Ewe	305	12.68
	Guan	61	2.54
	Mole-Dagbani	800	33.25
	Grusi	80	3.33
	Gurma	154	6.40
	Mande	27	1.12
	Other	67	2.78
Wealth index combined	Poorest	798	33.17
	Poorer	505	20.99
	Middle	471	19.58
	Richer	347	14.42
	Richest	285	11.85
Currently pregnant	No or unsure	2195	91.23
	Yes	211	8.77
Cooking fuel	Low pollution fuel	315	13.09
	Medium pollution fuel	694	28.84
	High pollution fuel	1397	58.06
Drinking water categories	Improved/clean	2103	87.41
	Unimproved/unclean	303	12.59
Toilet facilities categories	Improved/clean	1389	57.73
	Unimproved/unclean	1017	42.27

### Results of Chi-square tests of bivariate associations

Table 2 presents a bivariate analysis of anaemic status in a sample of 2406 individuals, examining various demographic variables such as age group, place of residence, region, educational level, religion, ethnicity, and wealth index. The table displays the distribution of individuals across different demographic categories by anaemic status, along with corresponding *p* values and chi-square test statistics indicating the association between each demographic variable and anaemic status. The results reveal statistically significant associations between anaemic status and age group, place of residence, region, education level, religion, ethnicity, wealth index, type of drinking water, toilet facility type, and cooking fuels. Additionally, the chi-square tests employed to address multicollinearity concerns identifed significant associations between wealth index and type of cooking fuels, wealth index and participant’s region, region and cooking fuels used, ethnicity and region, and religion and region. These findings guided the selection of variables for inclusion in the subsequent logistic regression models.

**Table 2:** Contingency table of anaemic status and covariates.

Variables	Levels/categories	Anaemic status		Total (*N* = 2406)	*p* value, Chi-square
		Non-anaemic (N = 1009)	Anaemic (N = 1397)		
Age in 5-year groups	15–19	28 (2.8%)	62 (4.4%)	90 (3.7%)	0.001,21.901
	20–24	152 (15.1%)	284 (20.3%)	436 (18.1%)	
	25–29	276 (27.4%)	364 (26.1%)	640 (26.6%)	
	30–34	239 (23.7%)	295 (21.1%)	534 (22.2%)	
	35–39	188 (18.6%)	243 (17.4%)	431 (17.9%)	
	40–44	103 (10.2%)	106 (7.6%)	209 (8.7%)	
	45–49	23 (2.3%)	43 (3.1%)	66 (2.7%)	
Place of residence	Urban	450 (44.6%)	454 (32.5%)	904 (37.6%)	< 0.001, 36.0575
	Rural	559 (55.4%)	943 (67.5%)	1502 (62.4%)	
Region	Western	117 (11.6%)	123 (8.8%)	240 (10.0%)	< 0.001,101.773
	Central	73 (7.2%)	141 (10.1%)	214 (8.9%)	
	Greater Accra	99 (9.8%)	73 (5.2%)	172 (7.1%)	
	Volta	98 (9.7%)	112 (8.0%)	210 (8.7%)	
	Eastern	99 (9.8%)	88 (6.3%)	187 (7.8%)	
	Ashanti	140 (13.9%)	107 (7.7%)	247 (10.3%)	
	Brong Ahafo	92 (9.1%)	138 (9.9%)	230 (9.6%)	
	Northern	128 (12.7%)	278 (19.9%)	406 (16.9%)	
	Upper East	64 (6.3%)	170 (12.2%)	234 (9.7%)	
	Upper West	99 (9.8%)	167 (12.0%)	266 (11.1%)	
Highest educational level	No education	235 (23.3%)	454 (32.5%)	689 (28.6%)	< 0.001, 48.853
	Primary	196 (19.4%)	328 (23.5%)	524 (21.8%)	
	Secondary	515 (51.0%)	573 (41.0%)	1088 (45.2%)	
	Higher	63 (6.2%)	42 (3.0%)	105 (4.4%)	
Religion	Christianity	779 (77.2%)	926 (66.3%)	1705 (70.9%)	< 0.001,36.1586
	Islam	193 (19.1%)	391 (28.0%)	584 (24.3%)	
	Traditional/spiritualist	19 (1.9%)	30 (2.1%)	49 (2.0%)	
	No religion	17 (1.7%)	49 (3.5%)	66 (2.7%)	
	Other	1 (0.1%)	1 (0.1%)	2 (0.1%)	
Ethnicity	Akan	393 (38.9%)	419 (30.0%)	812 (33.7%)	< 0.001,52.1017
	Ga/Dangme	52 (5.2%)	48 (3.4%)	100 (4.2%)	
	Ewe	146 (14.5%)	159 (11.4%)	305 (12.7%)	
	Guan	27 (2.7%)	34 (2.4%)	61 (2.5%)	
	Mole-Dagbani	266 (26.4%)	534 (38.2%)	800 (33.3%)	
	Grusi	34 (3.4%)	46 (3.3%)	80 (3.3%)	
	Gurma	56 (5.6%)	98 (7.0%)	154 (6.4%)	
	Mande	13 (1.3%)	14 (1.0%)	27 (1.1%)	
	Other	22 (2.2%)	45 (3.2%)	67 (2.8%)	
Wealth index combined	Poorest	248 (24.6%)	550 (39.4%)	798 (33.2%)	< 0.001,120.121
	Poorer	183 (18.1%)	322 (23.0%)	505 (21.0%)	
	Middle	212 (21.0%)	259 (18.5%)	471 (19.6%)	
	Richer	185 (18.3%)	162 (11.6%)	347 (14.4%)	
	Richest	181 (17.9%)	104 (7.4%)	285 (11.8%)	
Currently pregnant	No or unsure	914 (90.6%)	1281 (91.7%)	2195 (91.2%)	0.3797, 0.7714
	Yes	95 (9.4%)	116 (8.3%)	211 (8.8%)	
Cooking fuel	Low pollution fuel	185 (18.3%)	130 (9.3%)	315 (13.1%)	< 0.001,70.269
	Medium pollution fuel	330 (32.7%)	364 (26.1%)	694 (28.8%)	
	High pollution fuel	494 (49.0%)	903 (64.6%)	1397 (58.1%)	
Drinking water categories	Improved/clean	909 (90.1%)	1194 (85.5%)	2103 (87.4%)	< 0.001,10.9459
	Unimproved/unclean	100 (9.9%)	203 (14.5%)	303 (12.6%)	
Toilet facilities categories	Improved/clean	656 (65.0%)	733 (52.5%)	1389 (57.7%)	< 0.001,37.2728
	Unimproved/unclean	353 (35.0%)	664 (47.5%)	1017 (42.3%)	

### Results of the sequential binary logit analysis

Model 1 in Table 3 presents the results of a simple binary logit model, where the outcome variable is anaemia status, and the predictor variable is the type of cooking fuel used, with low pollution fuel as the reference category. The results suggest that the type of cooking fuel used is a significant predictor of getting anaemia, with medium and high pollution fuels being associated with higher odds of getting anaemia compared to low pollution fuel. More broadly, the odds ratio (OR) estimate for medium pollution fuel is 1.57, implying that the odds of contracting anaemia are 1.57 times higher for individuals using medium pollution fuel compared to those using low pollution fuel. Similarly, the OR for high-pollution fuel is 2.60, indicating that the odds of getting anaemia are 2.60 times higher for individuals using high-pollution fuel relative to those using low-pollution fuel.

**Table 3:** Tabulated results of the sequential binary logit analysis.

Variable	OR	*p* value	OR2.5%	OR97.5%
Model 1: Binary logistic regression results with only type of cooking fuel as covariate				
(Intercept)	0.703	0.0002	0.561	0.878
Cooking fuel (Ref = Low pollution fuel)				
Medium pollution fuel	1.57	0.001	1.20	2.05
High pollution fuel	2.60	< 0.001	2.03	3.34

Variable	AOR	*p* value	OR2.5%	OR97.5%
Model 2: Multiple binary logistic regression results with cooking fuels and other selected covariates				
(Intercept)	0.929	0.8001	0.528	1.657
Cooking fuel (Ref : Low pollution fuel)				
Medium pollution fuel	1.35	0.036	1.02	1.80
High pollution fuel	1.66	0.002	1.21	2.28
Age in 5-year groups (Ref: 15–19)				
20–24	0.89	0.637	0.540	1.480
25–29	0.67	0.075	0.410	1.090
30–34	0.64	0.05	0.390	1.050
35–39	0.63	0.061	0.380	1.050
40–44	0.48	0.006	0.270	0.830
45–49	0.81	0.674	0.400	1.650
Region (Ref. = Western)				
Central	1.86	0.002	1.260	2.730
Greater Accra	1.02	0.91	0.670	1.560
Volta	1.0088	0.964	0.691	1.472
Eastern	0.9	0.608	0.610	1.330
Ashanti	0.84	0.346	0.580	1.210
Brong Ahafo	1.31	0.156	0.900	1.910
Northern	1.8	< 0.001	1.280	2.540
Upper East	2.26	< 0.001	1.520	3.350
Upper West	1.35	0.117	0.930	1.950
Place of residence (Ref. = Rural)				
Urban	1.15	0.228	0.920	1.430
Drinking water category: (Ref. = Improved/clean)				
Unimproved/unclean	1.14	0.354	0.860	1.500
Toilet facility category (Ref. = Improved/clean)				
Unimproved/unclean	1.17	0.105	0.970	1.420

Model 2 in Table 3 presents the results of an adjusted multiple binary logit model with anaemia status as the outcome variable, and cooking fuel, age, region, place of residence, drinking water category, and toilet facility category as the predictor variables based on the bivariate and multicollinearity analysis previously discussed. After adjusting for other covariates, the results show that individuals who use medium pollution fuel are 1.35 times more likely to have anaemia than those who use low pollution fuel (*p* = 0.036), while individuals who use high pollution fuel are 1.66 times more likely to have anaemia than those who use low pollution fuel (*p* = 0.002).

Older age groups have a lower likelihood of anaemia compared to the reference group (15–19). Individuals in the age group 40–44 experience a 52% decrease in the likelihood of having anaemia as those in the reference group (*p* = 0.006). The results also indicate that individuals living in the Northern, Central, and Upper East regions are at higher risk of anaemia than those living in the Western region. Individuals in the Northern Region are 1.8 times more likely to have anaemia than those in the Western Region (*p* < 0.001). Similarly, individuals in the Central and Upper East regions are 1.86 (*p* = 0.002) and 2.26 (*p* < 0.001) times more likely to have anaemia than those in the Western Region, respectively. The place of residence, drinking water category, and toilet facility category were not found to be significantly associated with anaemia after adjusting for other variables.

## Discussions

We found a 58.06% prevalence of anaemia (mildly anaemic, moderately anaemic, and severely anaemic) within the sample. Anaemia prevalence within our sample is significantly higher than prevalence rates from past studies, such as Pathirathna et al.^26^ finding a 36.1% prevalence in Sri Lanka, Habyarimana et al.^44^ reporting a 19.2% prevalence in Rwanda, and Ford et al.^45^ and Pinto^46^ reporting a 23% prevalence rates in Nepal, and Timor-Leste respectively. The significantly higher anaemic condition in our sample compared with evidence from the literature, for instance, the age-standardized point prevalence of 23.18%^12^ is alarming. Age, nutritional deficiencies, inflammation, and air pollution must be addressed to lessen anaemia burden among WRA in Ghana. Advocacy and the encouragement of health promotion initiatives for anaemia reduction and clean cooking fuel adoption are a few of such interventions.

We found an association between the kind of cooking fuel used and the likelihood of anaemia, with more polluting fuels linked to a higher risk of anaemia. This supports the abundance of literature linking the usage of biomass fuel (polluting fuels) to anaemia in WRA^27–32^. The biological plausibility of the association between solid fuel and anaemia is still understudied, but it is believed that exposure to combustion by-products from solid fuel can reduce oxygen delivery to the tissues and cause systemic inflammation. Additionally, combustion emissions from cooking fuels, such as particulate matter, may cause oxidative stress in red blood cells, affecting their shape and deformability and ultimately leading to anaemia^31,32^. Finally, inorganic gases CO and SO2 induce systemic inflammation and oxidative damage to red blood cells because they are highly soluble gases that may diffuse across alveolar and capillary membranes and quickly enter the circulation.

Promoting the use of cleaner fuels must be prioritized while the mechanisms underlying the high prevalence of anaemia associated with gas fuel and solid fuel use undergo scientific evaluations. Age was associated with anaemia, with older age groups having a lower risk of anaemia. Pinto^46^ conducted an analysis of the association between using solid fuel and anaemia among WRA, 15–49 years old in Timor-Leste and found women in the age group of 35–49 were at greater risk of having anaemia compared to 15–35 group of age. Morsy and ALhays^47^ found that women aged 36–50 years were more at risk when compared to other younger age groups. This contrasts our finding where older age groups had a lower risk of anaemia, with those in the age range of 40–44 seeing a 52% reduction in the likelihood of having anaemia. One plausible explanation could be younger WRA focus more on household needs and abandon their personal needs, but there is the likelihood of other underlying factors that predict the lower odds of anaemia risk among older WRA. This should be investigated further for a clearer understanding.

We identified region of residence as a risk factor for being anaemic within our sample, by which residents of the Northern, Central, and Upper East regions have a higher risk of anaemia than residents of the Western region. A lower socioeconomic status could be the reason for such regional variances. The energy-ladder model stressed income and general socioeconomic conditions as a determinant of households’ move from unclean and ‘inferior’ fuels to clean and contemporary fuels^33,46^. This is true in light of the evidence produced by Bofah, Appiah-Konadu and Ngwu^48^, where the authors discovered that socioeconomic parameters such as income were an important driver of families’ preference for clean energy (LPG) as their primary cooking-energy source in Ghana. The Northern and Upper East regions remain one of the poorest regions in Ghana^49,50^, hence could fit into the energy-ladder model. Again, the Central Region of Ghana is one of the regions in Ghana where a large proportion of women make their living from fish smoking^51^. Preserving fish using smoke (most commonly from burning wood) may expose women to chronic smoke inhalation during the process, possibly increasing their risk of anaemia^52^. We however acknowledge that the above reasons may not be sufficient as there is the likelihood of other underlying factors to predict the higher odds of anaemia risk among WRA in the Northern, Central, and Upper East regions.

Ultimately, these results underline how crucial it is to use clean cooking fuels to lower the risk of anaemia, particularly in regions with higher levels of pollution. The findings also imply that age and place of residence should be taken into account in focused measures to reduce the prevalence of anaemia.

The findings call for actors within the energy and public health space in Ghana to strengthen efforts aimed at following the WHO recommendation on the use of cleaner fuels. These recommendations are (1) switching to alternative fuels (liquid petroleum gas (LPG), biogas, electricity and solar power); (2) creating better house ventilation including chimneys, smoke hoods, eaves spaces enlarged and cooking windows; and (3) avoiding the use of dried fuel wood for cooking. On the use of alternate fuels, Ghana’s effort has yielded just a 22% adoption rate^53^, leaving a huge deficit. This is particularly discouraging, in light of the country’s commitment to meeting SDG indicator 7.1.2 which seeks to increase the proportion of the population with primary reliance on clean fuels and technology. The Ministry of Health in Ghana, especially the public health division, should develop an awareness program that educates women, on the importance of considering alternative methods of using solid fuels or switching to clean fuels. This program must directly incorporate household heads since they wield decision-making power. This would expand their knowledge on IAP, especially from solid fuel, hence increasing the adoption of clean energy (ceteris paribus). In addition, increasing poverty alleviation efforts will go a long way toward aiding Ghana’s shift to cleaner cooking energy, especially in light of the plausibility we developed to explain the regional variance in anaemia risk.

## Conclusions

According to the findings of this study, anaemia is more likely to occur in WRA in Ghana who use high-pollutant fuels in their homes, such as wood, straw/shrubs/grass, agricultural crops, and animal dung. Thus, it is necessary to implement immediate initiatives to help minimise IAP, which has been linked to anaemia, particularly in WRA.

## Data Availability

The datasets used and/or analysed during the current study are available from the corresponding author upon reasonable request.

## Abbreviations

BMI: Body mass index
CO: Carbon monoxide
COPD: Chronic obstructive pulmonary disease
GBD: Global Burden of Disease
GDHS: Ghana Demographic and Health Survey
GSS: Ghana Statistical Service
HRQOL: Health-related quality of life
IAP: Indoor air pollution
IEA: International Energy Agency
LMICs: Lower- and middle-income countries
LPG: Liquefied petroleum gas
NO_2_: Nitrogen dioxide
SSA: Sub-Saharan Africa
SES: Socioeconomic status
WHO: World Health Organization
WRA: Women of reproductive age
